# Malignant hyperthermia as a rare cause of SIRS after cardiac surgery

**DOI:** 10.1186/1471-2253-14-S1-A10

**Published:** 2014-08-18

**Authors:** Nils Theuerkauf, Fritz Mellert, Ulf Guenther

**Affiliations:** 1 Department of Anesthesia and Critical Care, University Hospital of Bonn, Bonn, 53127, Germany

## Background

Use of extracorporal circulation (cardiopulmonary bypass) during cardiac surgery can cause a systemic inflammatory response. This so called „post-perfusion-syndrome“ (PPS) occurs in about a quarter of patients and results in clinical signs and symptoms of „systemic inflammatory response syndrome“ (SIRS) in 2-10% of patients. This condition is clinically associated with mild hyperthermia, acidosis, tachycardia and vasoplegia. It is generally treated with cristalloid infusions and vasopressors, and is mostly subsided by the next morning, at the latest after 48h.

Malignant hyperthermia is associated with a severe combined (respiratory and metabolic) acidosis, hyperlactatemia, hypercapnia, hyperthermia, grossly elevated serum levels of creatine kinase (CK) and acute renal failure.

## Case report

We present a 23-year old patient, who gradually developed multi-organ failure following mitral valve repair for severe secondary mitral regurgitation due to dilated cardiomyopathy. His past medical history included partial hepatectomy for hepatoblastoma in childhood (1982) and partial lung resection due to recurrent pneumothoraces in 2002. Induction of general anesthesia was carried out with thiopentone, sufentanil and cis-atracurium and was maintained with isoflurane as the hypnotic agent. Following surgery, the patient showed increased infusion requirements and was highly vasopressor-dependent. Due to oliguric renal failure and refractory metabolic acidosis, continuous veno-venous hemodiafiltration was instituted early. Increased volume and vasopressor needs gradually decreased within the first 30 hours. In conjunction with two episodes of body temperature peaks of > 39°C in „non-CRRT-intervals“, the deferred but disproportionate elevation of serum-creatinkinase activity led to the clinical suspicion of malignant hyperthermia (Table 1). Much later, long after hospital discharge, MH was confirmed by in vitro testing.

**Table 1:** Metabolic parameters and adrenergic drug application during the hospital stay of the patient. D=day; WBC=white blood count; CK=creatine kinase; TNI=troponine I; NA=noradrenalin, VP=vasopressin; DBX=dobutamin; ADR=adrenalin; MIL=milrinone

	D0	D1	D2	D3	D4	D7
Lactate (mmol/l)	10.2	3.8	2.5	1.5	1.5	1.0
pH	7.22	7.38	7.49	7.47	7.49	7.45
WBC (G/L)	28.91	20.12	18.33	19.71	27.93	19.53
CK (U/L)	1159	1645	17926	25119	19828	12908
CK-MB (µg/L)	67.4	65.9	168.7	62.2	26.1	4.4
TNI (ng/ml)	17.50	13.50	9.03	6.59	3.34	1.20
NA (µg/min)	130	40	50	5	-	-
VP (IU/h)	2	2	-	-	-	-
DBX (µg/min)	1000	1000	1000	1000	1000	1000
ADR (µg/min)	40	30	16	20	7	-
MIL (µg/h)	4000	4000	4000	4000	4000	-

## Conclusions

High volume requirements and vasopressor-dependency occur commonly following surgical mitral valve repair, especially in patients with severely impaired left ventricular function. Differential diagnosis of hyperthermia on the ICU includes microbial triggers of the systemic inflammatory process, which could have been the case in this patient, where an infection might have been suspected. PPS and SIRS do not prompt any suspicion of malignant hyperthermia. Nevertheless, rare causes of both, hyperthermia and volume-depletion should be taken into account, even if the clinical course is rather mild in a way so that MH appears almost unlikely.

